# Association between social capital, mental health, and digital health literacy among the university students in China: a multigroup analysis based on major difference

**DOI:** 10.1186/s12889-024-19672-7

**Published:** 2024-08-13

**Authors:** Jiajia Zhao, Limei Nie, Lutong Pan, Mingli Pang, Jieru Wang, Yue Zhou, Rui Chen, Hui Liu, Xixing Xu, Chengchao Zhou, Shixue Li, Fanlei Kong

**Affiliations:** 1 Centre for Health Management and Policy Research, School of Public Health, Cheeloo College of Medicine, Shandong University, Jinan, 250012 China; 2 NHC Key Lab of Health Economics and Policy Research, Shandong University, Jinan, 250012 China; 3 Institute of Health and Elderly Care, Shandong University, Jinan, Shandong China; 4 Department of Mathematics, College of Art and Science, New York University, New York, 10003 USA

**Keywords:** Digital health literacy, Social capital, Mental health, University students

## Abstract

**Background:**

This study aimed to clarify medical-nonmedical difference on the relationship between social capital, mental health and digital health literacy of university students in China, and furtherly provide evidence-based suggestions on the improvement of the digital health literacy for the university students.

**Methods:**

The snowball sampling method was used to collect data from the university students (including medical students and nonmedical students) through online questionnaires, and finally 1472 university students were included for the data analysis, of whom, 665 (45.18%) were medical students, 807 (54.82%) were nonmedical students; 462 (31.39%) were male, 1010 (68.61%) were female. Mean value of the age was 21.34 ± 2.33 for medical students vs. 20.96 ± 2.16 for nonmedical students. Descriptive analysis, chi-square test analysis, one-way Analysis of Variance (conducted by SPSS) and structural equation modeling (conducted by AMOS) were employed to explore the difference on the relationship between social capital, mental health and digital health literacy between the medical students and nonmedical students.

**Results:**

The mean value of the digital health literacy was 36.27 (37.33 for medical students vs. 35.39 for nonmedical students). The SEM analysis showed that there was a statistically positive correlation between social capital and digital health literacy (stronger among the nonmedical students (0.317) than medical students (0.184)). Mental health had a statistically positive impact on the digital health literacy among medical students (0.242), but statistically significant correlation was not observed in nonmedical students (0.017). Social capital was negatively correlated with the mental health for both medical students and NMS (stronger among the nonmedical students (0.366) than medical students (0.255)). And the fitness indices of SEM were same between medical students and nonmedical students (GFI = 0.911, AGFI = 0.859, CFI = 0.922, RMSEA = 0.074).

**Conclusion:**

The digital health literacy of the university student was relatively high. Both social capital and mental health could exert a positive effect on digital health literacy, while social capital was found to be positively associated with mental health. Statistical difference was found between medical students and nonmedical students on the above correlations. Implications were given on the improvement of the digital health literacy among university students in China.

## Introduction

As the development of the science and technology, the number of internet users had reached 1.067 billion by the end of 2023 in China [1], which made the internet become an important and useful source of health information [2]. However, previous literature reported that up to 1/3 of the medical misinformation were prevalent on the social media account [3], leading to incorrect healthcare behaviors and treatment [4, 5], which further lead to the aggravation and higher incidence of negative health outcomes of the individual [6]. Digital health literacy (DHL) refers to the skills to search, select, evaluate, and apply online health information and healthcare related digital applications [7, 8]. The difference of DHL between individuals could affect the health related behaviors (such as exercise and diets), and contributed to health inequalities [9, 10]. Sufficient level of DHL could improve the health status by promoting access and utilization of digital health information [11]. However, the previous studies about DHL mainly focused on the medical students (MS) or hadn't clarify the difference between MS and nonmedical students (NMS) [12, 13].

Social capital was divided into cognitive (e.g. reciprocity and support) and structural components (e.g. participation and connection) [14, 15]. Many researchers had demonstrated the relationship between social capital and eHealth literacy [16]. Cui et al. clarified the direct and positive association between social capital and eHealth literacy among Chinese elderly people [17], and Stellefson et al. found a significant relationship of social support on eHealth literacy among patients living with chronic obstructive pulmonary disease [18]. Moreover, Arcury et al. also clarified the potential benefits of social support in improving low eHealth literacy [19]. However, few existed studies had explored the relationship between social capital and digital health literacy.

Mental health is a state of mental well-being that enables people to cope with the stresses of life, realize their abilities, learn well and work well, and contribute to their community [20]. There is no unanimous conclusion on the relationship between mental health and eHealth literacy. Previous researches mainly showed the positive association between mental health and DHL [21]. However, the university students who had mental health problems sought health information more frequently online in Ethiopia [22], and the mental health were found to be improved by higher DHL in Vietnam [23]. There were few studies explored the impact of mental health on DHL by SEM.

Many researches demonstrated a positive relationship between social capital and mental health [24, 25], that is, individuals with lower social capital were less likely to be able to deal with psychological problems and were more likely to suffer from mental illnesses [26]. Backhaus et al. reported that students with lower social capital were at greater risk of developing symptoms of clinically relevant mental problems in 12 countries [27]. Moreover, higher social capital could lower the depression symptoms by relieving stress among multicultural adolescents in Korea [28], as well as improve the mental condition of Chinese university students [29].

**Fig. 1:**
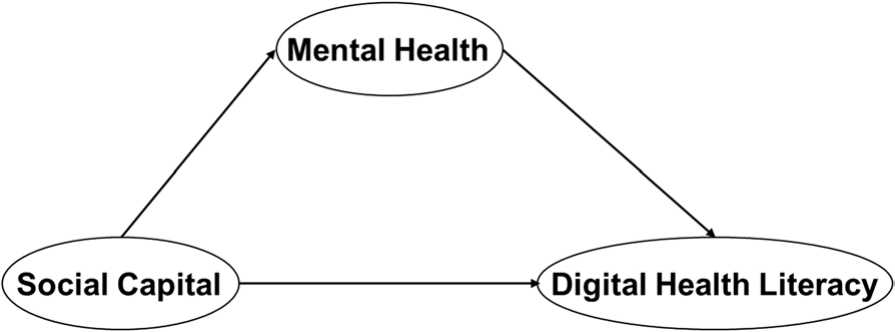
Conceptual model of the association between social capital and mental health and DHL

Some previous studies have explored the association between mental health and DHL, social capital and DHL, social capital and mental health, yet no research has ever clarified the relationship between social capital, mental health and DHL by using (SEM) among the university students in China. Thus, this study aimed to identify the relationship and further investigate whether there was medical-nonmedical difference between social capital, mental health and DHL among the university students in China. According to the research purpose above, three hypotheses were proposed to construct the hypothetical model for this study (as shown in Fig. 1). First, it was hypothesized that DHL was positively associated with social capital in university students(H1). Second, DHL was correlated with mental health among the university students(H2). Third, mental health was positively associated with social capital in university students(H3).

## Methods

### Study participants

#### Sample selection

The snowball sampling method was used to collect the data via an online platform (*Wenjuan.com*) among the university students in China. The content of questionnaire was verified through the pilot survey by using snowball sampling among 304 university students to ensure that the questions were understandable and appropriate. A total of 1527 samples were collected online from September 20 to October 24,2022. Of these, 1472 university students were finally included for analysis, of which 665 were MS, 807 were NMS. All respondents expressed their willingness to participate and understand the background and purpose of the study.

#### Sample characteristics

Table 1 summarized the general demographic characteristics of university students in this study. 1472 participants were included in the data analysis, of whom 462 (31.39%) were male, 1010 (68.61%) were female. There were more female than male in both MS (219 for male and 446 for female) and NMS (243 for male and 564 for female), as well as more university students lived in the urban (1019) than in the rural areas (453). The mean value of the age was 21.34 ± 2.33 for MS vs. 20.96 ± 2.16 for NMS, showing that MS were older than NMS. In terms of average monthly household income, about 2/5 of the subjects belonged to the group of 5000–10000 RMB (570, 38.72%). Regarding the average monthly spending, about 2/3 of the participants belonged to the group of 1000–2000 RMB (970, 65.90%). In terms of health status compared to last year, about 2/5 of the participants belonged to the same health status compared to the last year. Regarding to the health status compared to peers, more than half of the participants belonged to the same health status compared to peers. The mean value of the DHL was 36.27 (37.33 for MS vs. 35.39 for NMS), showing a high level of DHL among university students.

**Table 1:** Participants' descriptive characteristics

Variable	Major		χ2 Test/ANOVA
	**MS**	**NMS**	
	**n(%)/M**ean ± SD	**n(%)/M**ean ± SD	
Total	665(45.18%)	807(54.82%)	
Gender			
Male	219(47.40%)	243(52.60%)	1.347^a^ *P* = 0.246
Female	446(44.16%)	564(52.84%)	
Residence			
Urban	456(44.75%)	563(55.25%)	0.244^a^ *P* = 0.622
Rural	209(46.14%)	244(53.86%)	
Age	21.34 ± 2.33	20.96 ± 2.16	10.742^b^ *P* = 0.001
Average Monthly Household Income (RMB)			
0–3000	71(43.03%)	94(56.97%)	0.727^a^ *P* = 0.867
3000–5000	159(45.30%)	192(54.70%)	
5000–10000	264(46.32%)	306(53.68%)	
> 10,000	171(44.30%)	215(55.70%)	
Average Monthly Spending (RMB)			
0–1000	82(38.32%)	132(61.68%)	6.366^a^ *P* = 0.095
1000–2000	458(47.22%)	512(52.78%)	
2000–5000	108(44.08%)	137(55.92%)	
> 5000	17(39.53%)	26(60.47%)	
Health Compared to Last Year			
Much Worse	29(35.37%)	53(64.63%)	14.456^*a*^ *P* = 0.006
Worse	185(41.76%)	258(58.24%)	
About Same	320(49.92%)	321(50.08%)	
Better	102(40.64%)	149(59.36%)	
Much Better	29(52.73%)	26(47.27%)	
Health Compared to Peers			
Much Worse	16(34.04%)	31(65.96%)	10.531^*a*^ *P* = 0.032
Worse	129(40.19%)	192(59.81%)	
About Same	385(48.61%)	407(51.39%)	
Better	107(42.13%)	147(57.87%)	
Much Better	28(48.28%)	30(51.72%)	
DHL	37.33 ± 7.11	35.39 ± 7.21	26.807^b^ *P* < 0.001

*MS* Medical students, *NMS* Nonmedical students, *SD* Standard deviation, *DHL* Digital health literacy

^a^means χ^2^ Test

^b^means ANOVA

### Measurements

#### General demographics

In this study, general demographics included gender, residence, age, major, average monthly household income, average monthly spending, health status compare to peers, and health status compare to last year. The options for hukou were urban and rural, and the major were divided into medical and nonmedical university students. Average monthly household income was divided into below 3000 RMB, 3000–5000 RMB, 5000–10000 RMB, and more than 10,000 RMB. Average monthly spending were divided in below 1000 RMB,1000–2000 RMB, 2000–5000 RMB, and more than 5000 RMB. health status compared to peers and health status compared to last year were both categorized in five classes as much worse / worse / about same / better / much better.

#### DHL

DHL was measured by digital health literacy assessment (DHLA), which was designed by Peggy Liu in Taiwan China [30] and tested its reliability and validity in Mainland China by Nie et al. [31]. DHL was divided into two sections, named self-rated of digital health literacy (questions 1 to 6) and trust degree of online health information (questions 7 to 10) separately. The Cronbach's α of the questionnaire was 0.923 and show the good internal consistency. The total score of DHLA is between 10 to 50, with a higher score indicating a better DHL.

#### Social capital

Social capital was assessed by Chinese Shortened Social Capital Scale (CSSCS) designed by Xu et al. [32], which had been validated and used widely among Chinese population [32, 33]. The CSSCS includes two subscales: structural social capital and cognitive social capital. The structural social capital includes three dimensions: social participation (questions 1, 2, 3 and 4), social support (questions 5, 6, 7 and 8), and social connection (questions 9,10 and 11); the cognitive social capital includes three dimensions: trust (questions 12, 13 and 14), cohesion (questions 15, 16, 17, 18 and 19), and reciprocity (questions 20 21 and 22). The total score of CSSCS is between 22 to 110, with a higher score shows the better social capital. Considering the social fact of the university students (having no children), the 9th questions related to children was deleted from the original scale for this study. The Cronbach's α of the questionnaire was 0.882 after the deletion of the 9th question, while the Cronbach's α of each dimension (social participation, social support, social connection, trust, cohesion, and reciprocity) were 0.882, 0.719, 0.671,0.697, 0.851 and 0.796 separately, suggesting that the scale after deleting the 9th question had good internal consistency [34, 35]. Construct validity was tested to estimate the validity of CSSCS by correlation coefficient of each item of each dimension (social participation, social support, social connection, trust, cohesion, and reciprocity) respectively. Large effect sizes were observed in all dimensions (correlation coefficient ≥ 0.50) [35], indicating the feasibility of using CSSCS among the university students in this study.

#### Mental health

Mental health was assessed by Chinese version of Depression Anxiety Stress Scale (DASS-21), which had been validated by Wang et al. among Chinese university students and showed high reliability and validity [36]. Three dimensions (depression, anxiety and stress) were included in DASS-21. The depression scale included questions 3, 5, 10, 16, and 17, the anxiety scale included questions 2, 4, 7,9,15, 19 and 20, and the stress scale included questions 1, 6, 8, 11, 12, 14, 18 and 21. The DASS-21 is determined using a 4-point Likert scale: 1 = never, 2 = often, 3 = sometimes, 4 = always. The total score of DASS-21 is 21–84, and the higher scores indicating worse mental health. The Cronbach's α of DASS-21 was 0.962, of which show the good internal consistency.

### Statistical analysis

Descriptive statistics were used to show the general distribution of the basic demographics of the participants. The chi-square was used to compare the major difference in general demographics, and one-way Analysis of Variance (ANOVA) were used to compare the difference in social capital, mental health and DHL among MS and NMS in China. A *P* value < 0.05 denotes statistical significance, and all the analysis were executed using the Statistical Package for Social Science for Windows (SPSS, version 25.0, IBM, Armonk, New York, USA).

Structural Equation Modeling (SEM) was used to explore the association between the social capital, mental health and DHL of the university students in China. The maximum-likelihood estimation was used to predict the best-fitting model in the study. The SEM model consists two types of latent variables: exogenous variable and endogenous variable. The exogenous variable was social capital, and the endogenous variables were mental health and DHL. In the diagrams, regression and coefficients between variables were focused on standardized regression coefficients performed to interpret and compare. Assessment of the model fitness calculates how the theoretical model might be consistent with the empirical data. The chi-square test, goodness of fit index (GFI), adjusted goodness of fit index (AGFI), normed fit index (NFI), incremental fit index (IFI), comparative fitness index (CFI), root mean square error of approximation (RMSEA) and the Hoelter’s critical N (CN) were reported for analysis of model fitness, as in many previous studies. When sample sizes are large (as in the present study), a non-significant chi-square is rarely obtained [37–39]. GFI, AGFI, NFI, IFI, CFI, and RMSEA were instead adopted as the fitness indices in this study. The hypothetical models are regarded to be well fitted to the data when *P* > 0.05; GFI > 0.90, AGFI > 0.80, NFI > 0.90, IFI > 0.90,CFI > 0.90, and RMSEA < 0.08 [40, 41]. The CN values is higher than 200, indicating multi- group restriction models have the property of constancy and can be estimated across group categories [42].The change in RMSEA was used to assess the measurement invariance for the comparison of the less restricted model with the more constrained model. With a sample of more than 300, the change of CFI is less than 0.010 and the change of RMSEA (ΔRMSEA) is less than 0.015 implies that measurement invariance has been successfully established [43, 44]. The AMOS (version 28.0, IBM, Armonk, New York, USA) statistical software package for Windows was executed to measure the model's fitness indexes, and obtain the maximum-likelihood estimates of model parameters.

### Ethics approval and consent to participate

The survey and data were obtained with informed consent from all participants. The Ethics Committee of Shandong University reviewed and approved this study (task no. LL20220425) and was in accordance with the 1964 Helsinki declaration and its later amendments or comparable ethical standards.

## Results

### Structural model

#### Model fitness indices

**Fig. 2:**
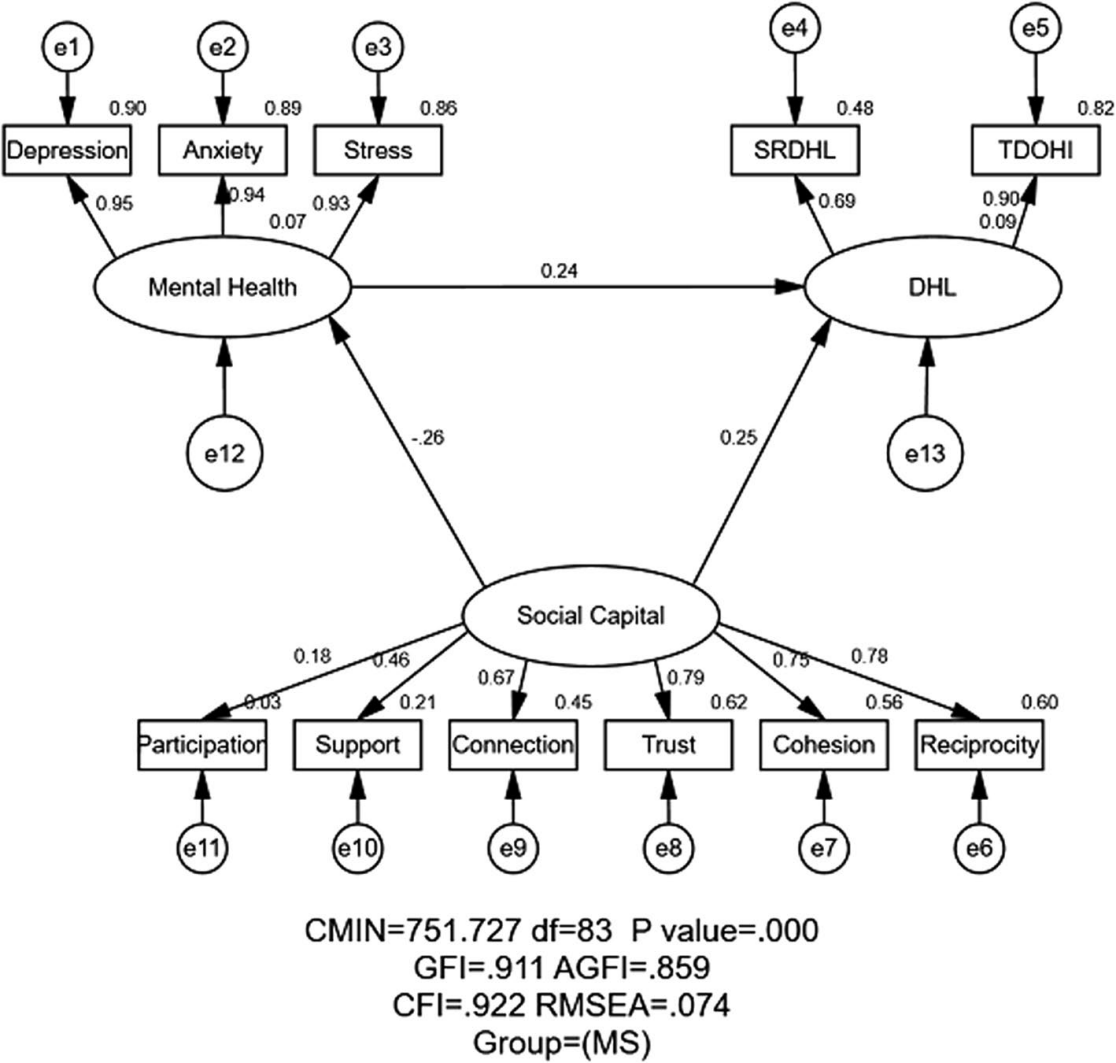
Structural equation modeling analysis of the association between social capital, mental health and DHL (digital health literacy) of the MS (*n* = 665). Employing the data, association between social capital, mental health and DHL were analyzed. The arrows indicate the associations and directions between variables. All parameter estimates were statistically significant (*P* < 0.001). Note: CMIN = Chi Square; df = Degrees of Freedom; GFI = Goodness of Fit Index; AGFI = Adjusted Goodness of Fit Index; CFI = Comparative Fitness Index; RMSEA = Root-mean Square; MS = Medical Students; SRDHL = Self-rated of Digital Health Literacy; TDOHI = Trust Degree of Online Health Information

**Fig. 3:**
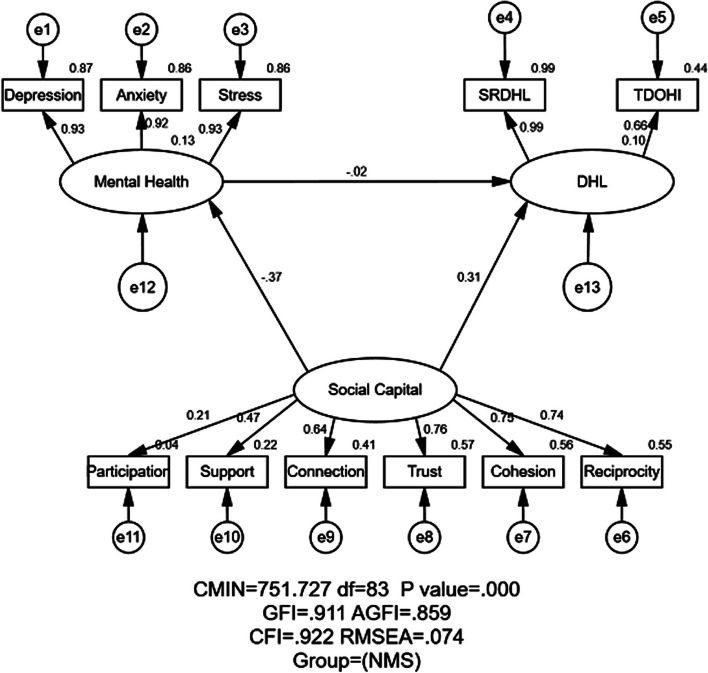
Structural equation modeling analysis of the association between social capital, mental health and DHL (digital health literacy) of the NMS (*n* = 807). Employing the data, association between social capital, mental health and DHL were analyzed. The arrows indicate the associations and directions between variables. All parameter estimates were statistically significant (*P* < 0.001), except for the impact of mental health on DHL. Note: CMIN = Chi Square; df = Degrees of Freedom; GFI = Goodness of Fit Index; AGFI = Adjusted Goodness of Fit Index; CFI = Comparative Fitness Index; RMSEA = Root-mean Square; NMS = Nonmedical Students; SRDHL = Self-rated of Digital Health Literacy; TDOHI = Trust Degree of Online Health Information

Figures 2 and 3 displayed the unconstrained model of MS and NMS groups, respectively. As shown in Figs. 2 and 3, the estimates of model fitness were the same for the two groups in M1 and M2: GFI = 0.911 > 0.90, AGFI = 0.859 > 0.80, NFI = 0.913, IFI = 0.922, CFI = 0.922 > 0.90, and RMSEA = 0.074 < 0.08, indicating the proposed model fit the empirical data well for both MS and NMS. The chi-square in AMOS was called CMIN and it was significant (*P* < 0.001) in this study. However, the CMIN was not applied to appraise the fitness index in the current study due to it is sensitive to the sample size [45].

#### Association between social capital, mental health and DHL assessed by SEM

Table 2, Figs. 2 and 3 illustrated the association of standardized effects between social capital, mental health and DHL. The total effects in the path mode consisted of direct and indirect. In Figs. 2 and 3, social capital → DHL showed the direct effect from social capital to DHL. And social capital → mental health → DHL showed the indirect effect of social capital on DHL via mental health.

**Table 2:** Standardized effects between social capital, mental health and DHL by major

Variable	Direct		Indirect		Total	
	**MS**	**NMS**	**MS**	**NMS**	**MS**	**NMS**
Social Capital → DHL	0.245	0.311	-0.062	0.060	0.184	0.371
Mental Health → DHL	0.242	-0.017	-	-	0.242	-0.017
Social Capital → Mental Health	-0.255	-0.366	-	-	-0.255	-0.366

*MS* Medical students, *NMS* Nonmedical students, *DHL* Digital health literacy

#### Relationship between social capital and DHL

As shown in Table 2, Figs. 2 and 3, social capital exerted both direct and indirect (through mental health) impact on DHL. The positive and direct effect of social capital on DHL were reported in both MS (standardized direct effects = 0.245) and NMS (standardized direct effects = 0.311). Meanwhile, the indirect effect of social capital on DHL through mental health in MS was negative (standardized indirect effects = − 0.062) but in NMS was no statically significance (standardized indirect effects = 0.060). Eventually, social capital exerted a stronger positive effect on DHL for NMS than MS (standardized total effects = 0.371 for NMS; standardized total effects = 0.184 for MS), implying that the higher social capital groups would have higher DHL.

#### Relationship between mental health and DHL

Mental health exerted positive indirect effect among MS (standardized total effects = 0.242) and negatively indirect effect (standardized total effects = − 0.017) among NMS on DHL, yet none statistical significance was found for NMS. As motioned in the method, the higher score of mental health implied the poorer mental health. In this study, the poorer mental health status of MS would indicate the higher DHL of MS, yet the correlation for NMS had no statistical significance.

#### Relationship between social capital and mental health

Social capital exerted negative and direct impact on mental health for both the MS and NMS (standardized total effect = − 0.255 for MS; standardized total effect = − 0.236 for NMS). Taking the higher score of mental health implied the poorer mental health, the higher level of social capital would indicate the better mental health for both MS and NMS in this study.

#### Measurement invariance across major

The authors used Harman's single-factor test for Common Method bias(CMB) [46], and the results showed that a total of 8 factors with eigenvalues > 1 were extracted, and the explanation rate of the first factor was 25.60%, which was lower than the 40% critical value, indicating that there was no common method bias in the data of this study [47].

Table 3 showed the results of related fitness indicators regarding the measurement invariance across majors and the fitness indexes in eight different models. To check whether the major was suitable for group comparison, the fitness indexes between MS and NMS should be compared first.

**Table 3:** Model invariance test using multi-group nested analysis

Model	χ2	df	χ2 /df	GFI	AGFI	NFI	IFI	CFI	RMSEA	ΔCFI	ΔRMSEA	CN
M1	751.727^a^	83	9.057	0.911	0.859	0.913	0.922	0.922	0.074	**-**	**-**	207
M2	751.727^a^	83	9.057	0.911	0.859	0.913	0.922	0.922	0.074	0	0	207
M3	751.727^a^	83	9.057	0.911	0.859	0.913	0.922	0.922	0.074	0	0	207
M4	755.970^a^	90	8.400	0.911	0.869	0.909	0.923	0.922	0.071	0	0.003	222
M5	793.876^a^	93	8.536	0.907	0.868	0.909	0.918	0.918	0.072	0.004	0.001	217
M6	793.877^a^	94	8.445	0.907	0.870	0.908	0.919	0.918	0.071	0	0.001	219
M7	800.877^a^	96	8.342	0.907	0.872	0.901	0.918	0.918	0.071	0	0	222

*M1* Medical students, *M2* Nonmedical students, *M3* Unconstrained, *M4* Measurement weights, *M5* Structural weights, *M6* Structural covariance, *M7* Structural residuals, *χ2 *Chi square, *df* Degrees of freedom, *GFI* Goodness of fit index, *AGFI* Adjusted goodness of fit index, *NFI* Normed fit index, *IFI* Incremental fit index, *CFI* Comparative fitness index, *RMSEA* Root-mean square error of approximation, ∆*CFI* Change of CFI, ∆*RMSEA* Change of RMSEA, *CN* Hoelter’s critical N

As illustrated in Table 3, the fitness indices of MS and NMS were same (GFI = 0.911, AGFI = 0.859, NFI = 0.913, IFI = 0.922, CFI = 0.922, RMSEA = 0.074 for both M1 and M2). The GFI, NFI, IFI and CFI were higher than 0.9, the AGFI was more than 0.8, and the RMSEA values were less than 0.08, implying the measurement invariance between two groups on the other models could be furtherly explored.

Then the ΔCFI and ΔRMSEA between M3 (unconstrained model), M4 (measurement weights model), M5 (structural weights model), M6 (structural covariance model) and M7 (structural residuals model) were used to evaluate the measurement invariance. The M3 does not restrict any model coefficient, the M4 assumes that the indicator loadings for the corresponding construct of each group are equal, while M5 constrains both the indicator loadings of the corresponding construct and the structural coefficients between two groups. Instead, M6 assumes the indicator loadings of the corresponding construct and the structural coefficients between two groups are equal. In addition, the indicator loadings, structural coefficients, endogenous variables' covariance, and variance of the exogenous variable between the groups are assumed equal in M7.

In Table 3, the ΔCFI was 0.004 between M4 and M5, and 0 between M3 and M4, M5 and M6, M6 and M7; the ΔRMSEA between M3 and M4, M4 and M5, M5 and M6, and M6 and M7, are 0.003, 0.001, 0.001 and 0, respectively. All the ΔCFI values were less than 0.010, implying that the measurement invariance of each model was established. Regarding ΔRMSEA, the value less than 0.015 established the measurement invariance between the M1, M2, M3, M4, M5, M6 and M7 models across the group of medical and NMS. In terms of the CN at the 0.05 significance level, all models had CN values greater than 200. Therefore, the above multi- group restriction models have the property of constancy and can be estimated across group categories.

## Discussion

### Principal findings

Using SEM, this study clarified the status and determinants of DHL among the university students in China, and investigated the major difference in social capital, mental health and DHL between MS and NMS. The findings of this study confirmed the first hypothesis, which hypothesized that DHL was positively associated with social capital in university students(H1). As for the second hypothesis, which hypothesized the DHL was correlated with mental health among the university students(H2) was only partially confirmed. Mental health was positively associated with social capital in university students(H3) was also confirmed in this study.

### Major difference in demographic characteristics

In terms of influencing effects of major among demographic characteristics, there were significant major difference among age, health compared to last year, and health compared to peers. MS were older than NMS in this study, which may due to the fact that the duration for most medical professions in China were longer than non-medical professions, which was similar with the research in United States and Canada [48, 49]. Regarding the health compared to last year and peers, MS suggested their health status become worse than last year and peers. It was similar with previous studies that MS have more negative attitudes towards their health status than NMS because of academic pressure [50, 51].

### DHL of the participants

The mean value of DHL was 36.27 for university students, implying the levels of DHL to be satisfactory. There was limited literature regarding levels of DHL and number of assessments used to measure DHL, which made it difficult to compare studies directly. Compared to peers, a study showed a similar result among university students in Denmark and Australia [52, 53], and findings from the Portuguese survey also reported the adequate DHL [54]. In this study, MS had higher scores of DHL than NMS, which contradicted previous research undertaken in university students in England [55] and Taiwan province of China [56].

### Social capital and DHL

Consistent with the first hypothesis(H1), positive effect of social capital on DHL was found in this study, which was similar with Paige's study conducted among the university students in America [57], and Wu et al. also clarified the positive association between social support and eHealth literacy [58]. However, Wang et al. proposed the self-realization of benefits of online information was more effective than social capital [59]. As for major difference, NMS exerted stronger effect than MS in this study, this may due to the fact that MS generally have better health knowledge than NMS and the social capital is not the main access to health information for them [60, 61]. Lee suggested that social capital was a resource, which had a positive effect in improving the efficiency of health information dissemination [62]. And Tsahi Zack Hayat clarified that social capital could help bridge the gap for individuals with lower levels of DHL [63]. Therefore, it will be important for university students, especially NMS, to improve the social capital to enhance the DHL.

### Mental health and DHL

Negative association between mental health and DHL was clarified among MS in present study (higher mental health score means lower mental health status by using DASS-21), which contradicted with the findings of Ning et al. [64]. And the relationship for NMS had no statistical significance. In other words, the lower mental health status would generally indicate the higher DHL. The second hypothesis(H2) was only partially confirmed. Previous studies showed MS had worse mental health than NMS [65–67].This might because MS mainly have a deeper perception and understanding of changes in mental health [68–70], they were more proactive in accessing health information and have higher DHL when they feel their mental health deteriorates. On the contrary, NMS were not able to detect and accurately describe the changes of mental health status to search this information online which may explain why the relationship between mental health and DHL for the NMS had no statistical significance. Moreover, MS might be worried about jeopardizing their expertise, which pushed them to search the informal sources (such as Internet) for health information when needed rather than formal healthcare-seeking behavior [71–74]. Young people with high knowledge were more likely to seek professional and useful mental health information online [75]. As for NMS with poor health knowledge, they were more willing to seek formal professional health services offline [76], rather than searching online. This implies the importance of improving basic medical language for NMS should be addressed. Existing studies and European initiatives advocated the digital health on education implementation among university students [77]. On the contrast of this study, Martin Baumgartner et al. focus on the improvement of digital health education in MS [78]. In a word, policymakers should add common digital health information in general health education among university students.

### Social capital and mental health

The third hypothesis(H3) regarded that positive correlation was determined in present study between social capital and mental health score both for MS and NMS, which was confirmed by the findings. It was in accordance with previous studies [79, 80]. That is to say, the higher social capital would generally imply the higher mental health status (higher mental health score means lower mental health status by using DASS-21). Social capital might protect mental health by playing a buffering role against mental health problems [81]. Meanwhile, social capital may enhance resilience to maintain better mental health [82]. Regarding the major difference between social capital and mental health, MS have stronger correlation than NMS. MS faced heavy academic schedules, disrupted work-life balance [83], while NMS had more spare time to socialise and relax. That is to say, NMS may have more opportunities to release negative emotions and stress through the buffering effect of social support and other entertainment ways. This suggested that the improvement of social support capital would be beneficial to mental health among university students.

### Implications

Firstly, the effect of social capital on mental health and DHL implied more attention should be paid to university students' social network, especially for the NMS with less health knowledge. Secondly, the statistical correlation between mental health and DHL highlighted the importance of maintenance and promotion of mental health for the university students, particularly in health consulting services and health education. Thirdly, mental health, a mediator of social capital and DHL, could affect the correlation between social capital and mental health. Therefore, the importance of mental health promotion among university students should be addressed. It is essential to accommodate the diversity of major and improve the health condition by enhancing the DHL of university students. The results of the current study on the empirical relationship between social capital, mental health and DHL could be useful in the development of fostering DHL among university students. These findings may provide evidence-based reference for improving DHL of university students in China.

## Limitations

There were several limitations of the present study should be addressed. First, information was collected by processing a self-assessment typical for online questionnaires, which may cause issues such as the uncertainty of the respondents' attitude and seriousness when completing it. Second, the cross-sectional nature of the data limits the study of causal relationships between these factors. Third, the total number of the female were almost twice as male may affect the results of the study. Forth, the 9th question of CSSCS scale was deleted. Despite the good results of the scale's reliability, the appropriateness of using the scale's original scoring method to calculate the total score of CSSCS needs to be further explored. Finally, although the snowball sampling could be utilized to reach the participants who may not be easily accessible, it may also bring potential bias stemming from the non-random selection of participants and the risk of oversampling certain subgroups within the population. Combining snowball sampling with other sampling methods or employing rigorous data analysis techniques to mitigate potential bias and fully representative of the target population are needed for the future researches.

## Conclusion

Despite the limitations above, the present study clarified the empirical association between social capital, mental health, and DHL among university students in China and highlight the major difference between MS and NMS. Social capital was found to be significantly and positively associated with mental health and DHL, with NMS slightly stronger than MS. Statistically significant positive association between mental health and DHL was found among MS, but not found among the NMS. The current study was a cross-sectional survey and future longitudinal studies should also be conducted to explore causal relationships. In addition, health includes physical health and social health in addition to mental health, and more research in the future will explore health and social capital and DHL in these categories. Since only the major difference were explored in this study, more researches focusing in difference of rural, other majors (engineering and non-engineering) etc. are worth to explore. Additionally, MS perceived themselves to be in poorer health than NMS. Therefore, a future study is planned to clarify the association between physical health, mental health, and the DHL among MS and NMS.

## Data Availability

Under reasonable requirements, the data and material of this study can be obtained from the corresponding author. The data are not publicly available due to privacy restrictions.

## Abbreviations

DHL: Digital Health Literacy
MS: Medical Students
NMS: Nonmedical Students
CSSCS: Chinese Shortened Social Capital Scale
DASS-21: Depression Anxiety Stress Scale
DHLA: Digital Health Literacy Assessment
ANOVA: One-way Analysis of Variance
SEM: Structural equation modeling
SRDHL: Self-rated Digital Health Literacy
TDOHI: Trust Degree of Online Health Information
CMIN: Chi square
RMSEA: Root mean square error of approximation
GFI: Goodness-of-fit index
AGFI: Adjusted goodness of fit Index
CFI: Comparative fit index
NFI: Normed Fit Index
IFI: Incremental Fit Index
CN: Hoelter’s critical N
CMB: Common Method bias
